# Transcutaneous electrical acupoint stimulation for the prevention of postoperative delirium in elderly surgical patients: A systematic review and meta-analysis

**DOI:** 10.3389/fnagi.2023.1046754

**Published:** 2023-01-31

**Authors:** Kai-Yu Huang, Shuang Liang, Lei Chen, Yong-Yi Xu, Antoine Grellet

**Affiliations:** ^1^Department of Acupuncture, Ningbo Hospital of Traditional Chinese Medicine, Affiliated Hospital of Zhejiang Chinese Medical University, Ningbo, China; ^2^The First Clinical Medical College, Nanjing University of Chinese Medicine, Nanjing, China

**Keywords:** transcutaneous electrical acupoint stimulation, postoperative delirium, elderly patient, surgery, systematic review, meta-analysis

## Abstract

**Objective:**

This systematic review and meta-analysis aimed to evaluate the preventive effect of transcutaneous electrical acupoint stimulation on postoperative delirium in elderly surgical patients.

**Methods:**

PubMed, CENTRAL, China National Knowledge Infrastructure, and WanFang databases were searched for randomized controlled trials regarding the effect of transcutaneous electrical acupoint stimulation on preventing postoperative delirium in elderly patients undergoing any type of surgery. The primary outcome was the incidence of postoperative delirium. The secondary outcome was the duration of postoperative delirium. All analyses were conducted using RevMan 5.3 and Stata 13.0 software.

**Results:**

Twelve trials with 991 participants were included, and most of them were at high/unclear risk of bias. Meta-analysis showed transcutaneous electrical acupoint stimulation could reduce the incidence of postoperative delirium (RR = 0.40, 95%CI = 0.29 to 0.55, *p* < 0.00001) and shorten the duration of postoperative delirium (MD = –0.97 days, 95%CI = −1.72 to −0.22, *p* = 0.01). Subgroup analyses demonstrated that transcutaneous electrical acupoint stimulation reduced the incidence of postoperative delirium in elderly patients undergoing orthopedic surgery and thoracic surgery, but not digestive surgery; transcutaneous electrical acupoint stimulation with dilatational wave and with continuous wave were both beneficial; and transcutaneous electrical acupoint stimulation was favored when compared to blank and sham control.

**Conclusion:**

Transcutaneous electrical acupoint stimulation could reduce the incidence of postoperative delirium and shorten the duration of postoperative delirium in elderly surgical patients. The findings should be interpreted with caution due to weak evidence. High-quality, large sample, and multi-center trials are needed to further confirm the preliminary findings.

**Systematic review registration**: https://inplasy.com/inplasy-2022-7-0096/, identifier: INPLASY202270096.

## 1. Introduction

Postoperative delirium (POD) is a relatively common complication, which mainly occurs within 24–72 h after surgery and is characterized by acute and fluctuating disturbances in attention, awareness, and cognition (Oh and Park, 2019). In general, POD is more common in elderly patients (Gracie et al., 2021). The incidence of POD is 20–27% in patients older than 60 years, and increases by age (Avidan et al., 2017). The incidence is also correlated with type of surgery, generally lower after minor surgery, and higher after major and emergency surgery (Yamagata et al., 2005). The occurrence of POD is associated with extension of hospitalization time, increase of hospitalization expense, long-term cognitive dysfunction, and increased risk of mortality (Li et al., 2022). Therefore, POD is a huge burden on elderly surgical patients’ health and the healthcare system as a whole.

Currently, treatment options for POD do not appear to reduce related mortality and morbidity (Méndez-Martínez et al., 2021). It is essential for clinicians to appropriately adjust perioperative care plans for primary prevention. Avoiding perioperative polypharmacy, depth of anesthesia monitoring, and postoperative dexamethasone are frequently-adopted POD risk reducing interventions (Vlisides and Avidan, 2019; Jin et al., 2020). While because of the incomplete pathophysiologic understanding and limited guideline evidence, the prevention of POD in elderly surgical patients is full of challenges (Duning et al., 2021).

As a non-invasive treatment, transcutaneous electrical acupoint stimulation (TEAS) combines the advantages of acupuncture and transcutaneous electrical nerve stimulation by pasting surface electrodes on target acupoints and then imposing electrical stimulation (Chiou et al., 2020; Chen et al., 2021). TEAS has been used widely for the prevention of common postoperative complications, such as postoperative cognitive dysfunction (Zhang et al., 2021), postoperative nausea and vomiting (Chen et al., 2020), and so on. In recent years, an increasing number of trials have been published to support the effectiveness of TEAS for the prevention of POD in elderly patients in terms of reducing the incidence of POD and shortening the duration of POD (Yang et al., 2017; Fan et al., 2022).

So far, no meta-analysis has been published to evaluate the preventive effect of TEAS on POD in elderly surgical patients. In view of this, we performed a systematic review and meta-analysis to investigate the effect of TEAS on POD in elderly surgical patients, which might provide more effective and accurate strategies for POD in clinical practice.

## 2. Materials and methods

### 2.1. Protocol and registration

The current review was performed following the recommendation of the Cochrane Handbook for Systematic Reviews of Interventions (Higgins et al., 2019) and is reported in compliance with the Preferred Reporting Items for Systematic Reviews and Meta-Analyses (PRISMA) 2020 statement: an updated guideline for reporting systematic reviews (Page et al., 2021). The protocol was registered with INPLASY (registration number INPLASY202270096).

### 2.2. Search strategy

A recent meta-epidemiological study showed that the combined retrieval power of PubMed, CENTRAL, China National Knowledge Infrastructure (CNKI), and WanFang databases was considered an efficient choice to retrieve acupuncture randomized controlled trials (RCTs) (Guo et al., 2022). So, we searched these four key databases for relevant RCTs about the effect of TEAS on preventing POD in elderly surgical patients, from the inception to July 31, 2022. The following terms were used in search strategies: (“transcutaneous electrical acupoint stimulation” OR “TEAS” OR “transcutaneous acupoint electrical stimulation” OR “TAES” OR “acustimulation” OR “acupuncture point” OR “acupoint” OR “acupuncture” OR “electroacupuncture” OR “electric”) AND (“postoperative delirium” OR “delirium” OR “delirium episodes” OR “acute brain syndrome”). The detailed search strategy is described in Supplementary Appendix 1. We also checked the reference lists of retrieved studies to identify other potentially eligible trials for inclusion.

### 2.3. Eligibility criteria

Studies were considered to be included if they met the following criteria: (1) population: elderly patients (age ≥ 60 years old) undergoing any type of surgery; (2) intervention: TEAS; (3) comparison: blank control, sham-stimulation therapy, or usual care; (4) outcome: POD; and (5) design: RCTs. The primary outcome was the incidence of POD, and the secondary outcome was the duration of POD. For duplicate studies, only the latest publication was included.

### 2.4. Study selection and data extraction

Two reviewers (K-YH and SL) independently searched the databases and evaluated eligible articles for inclusion. Disagreement was resolved by discussion with a third reviewer (LC). The following information was extracted independently by the reviewers (K-YH and SL): author’s name, publication year, sample size of two groups, type of surgery, details of TEAS (specific acupoints selection, waveform, frequency and intervention time) and control intervention, delirium assessment methods, and outcome measures.

### 2.5. Assessment of risk of bias

Two reviews (K-YH and SL) independently used the Cochrane Collaboration tool to assess the risk of bias of the selected trials (Higgins et al., 2019). The contents included random sequence generation, allocation concealment, blinding of participants and personnel, blinding of outcome assessment, incomplete outcome data, selective reporting, and other bias. These items were classified as “low risk,” “high risk,” or “unclear risk.” Disagreements were analyzed by the third reviewer (LC).

### 2.6. Statistical analysis

All analyses were conducted using RevMan 5.3 and Stata 13.0 software. Risk ratio (RR) with 95% confidence interval (CI) was used for dichotomous outcomes. Mean difference (MD) with 95% CI was used for continuous outcomes. Heterogeneity was examined using the *I^2^* test (Higgins and Thompson, 2002). Meta-analyses were conducted using a random-effects model regardless of heterogeneity. Two-tailed *p* < 0.05 were considered statistically significant. Subgroup analyses were performed based on different types of surgery (orthopedic, thoracic, and digestive surgery), TEAS waveform (dilatational and continuous wave), and control methods (blank control and sham TEAS). If sufficient trials (≥10 trials) were included, publication bias was assessed by visual inspection of the funnel plot and the formal Egger’s test (Egger et al., 1997).

## 3. Results

### 3.1. Study selection

**Figure 1:**
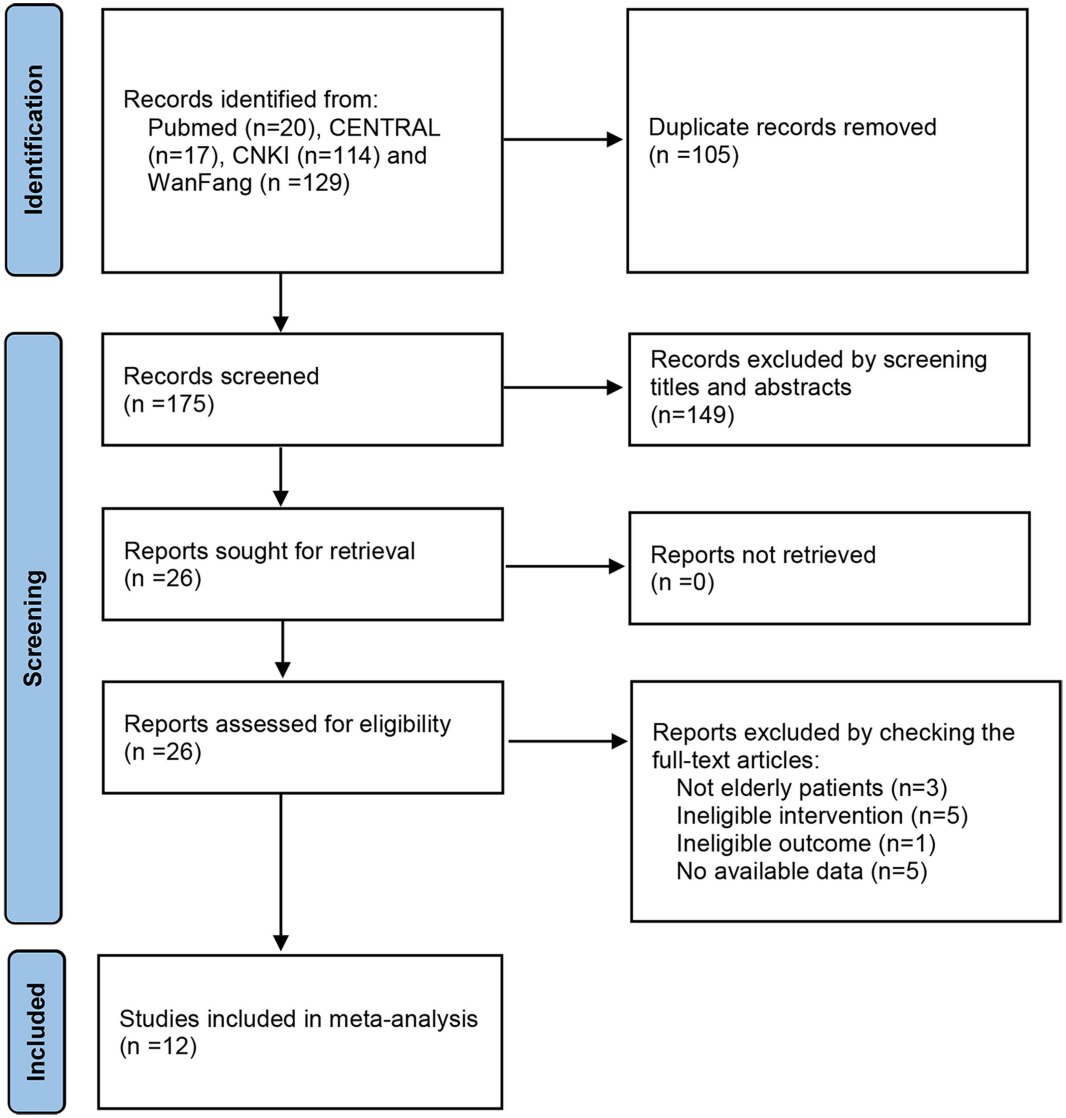
Flow diagram of the study selection process.

A total of 280 citations were identified from electronic databases. The number of citations decreased to 175 after duplicates were removed. By screening titles and abstracts, we excluded 149 studies. Of the 26 potentially eligible studies, 14 articles were excluded because of non-elderly patients, ineligible intervention, ineligible outcome and no available data. Finally, 12 trials were included (Gao et al., 2018; Qian et al., 2018; Liu, 2019; Ding et al., 2020, 2022; He and Sheng, 2020; Guo et al., 2021; Wei et al., 2021, 2022; Wu et al., 2021; Du et al., 2022; Xu et al., 2022). A flow diagram of study selection is displayed in Figure 1.

### 3.2. Study characteristics

Twelve trials with 991 participants were included in this review, with 511 in the TEAS group and 480 in the control group. These trials were published from 2018 to 2022. Eight trials reported patients who underwent orthopedic surgery (Gao et al., 2018; Qian et al., 2018; Ding et al., 2020, 2022; He and Sheng, 2020; Wei et al., 2021, 2022; Xu et al., 2022), three involved patients undergoing thoracic surgery (Liu, 2019; Guo et al., 2021; Du et al., 2022), and the remaining one had patients for digestive surgery (Wu et al., 2021). As assessment methods for POD, confusion assessment method (CAM) (Qian et al., 2018; Ding et al., 2020, 2022; He and Sheng, 2020; Wei et al., 2021; Wu et al., 2021), confusion assessment method-Chinese version (CAM-CR) (Wei et al., 2022), confusion assessment method for the intensive care unit (CAM-ICU) (Gao et al., 2018; Liu, 2019) and 4 A’s test (4AT) (Xu et al., 2022) were employed. All trials reported the incidence of POD, and four reported the duration of POD (Qian et al., 2018; Liu, 2019; He and Sheng, 2020; Guo et al., 2021). The characteristics of the included trials are listed in Table 1.

**Table 1:** Characteristics of the included studies.

Study	Sample size (TEAS/Control)	Type of surgery	TEAS group	Control group	Delirium assessment	Outcomes
He and Sheng (2020)	48 (24/24)	Orthopedic surgery	TEAS; LI4, PC6; continuous wave, 2 Hz; 30 min before anesthesia, stimulation for 30 min	Blank control	CAM	Incidence of POD; duration of POD
Qian et al. (2018)	60 (30/30)	Orthopedic surgery	TEAS; LI4, PC6; continuous wave, 2 Hz; 30 min before anesthesia, stimulation for 30 min	Blank control	CAM	Incidence of POD; duration of POD
Wei et al. (2022)	83 (41/42)	Orthopedic surgery	TEAS; GV29, PC6, LI11, PC7, PC8, GV20; dilatational wave, 2/6 Hz; 30 min before anesthesia till the end of surgery	Blank control	CAM-CR	Incidence of POD
Liu (2019)	118 (59/59)	Thoracic surgery	TEAS; LI4, PC6; dilatational wave, 2/100 Hz; from anesthesia to the end of surgery	Sham TEAS	CAM-ICU	Incidence of POD; duration of POD
Du et al. (2022)	140 (70/70)	Thoracic surgery	TEAS; ST36, SP10, PC6, LI4; dilatational wave, 2/100 Hz; from anesthesia to the end of surgery	Sham TEAS	Unclear	Incidence of POD
Xu et al. (2022)	70 (35/35)	Orthopedic surgery	TEAS; LI4, PC6, ST36; dilatational wave, 2/30 Hz; 30 min before anesthesia till the end of surgery	Blank control	4AT	Incidence of POD
Ding et al. (2020)	96 (48/48)	Orthopedic surgery	TEAS; GV20, GV24, LI4, PC6; dilatational wave, 2/30 Hz; 30 min before anesthesia till the end of surgery	Sham TEAS	CAM	Incidence of POD
Ding et al. (2022)	90 (30/30/30)	Orthopedic surgery	Preoperative TEAS (group A) or intraoperative TEAS (group B); GV20, GV24, LI4, PC6; dilatational wave, 2/100 Hz; 1 day before surgery and 30 min before the induction of anesthesia, stimulation for 30 min each time (group A) or from anesthesia to the end of surgery (group B)	Sham TEAS	CAM	Incidence of POD
Gao et al. (2018)	64 (32/32)	Orthopedic surgery	TEAS; LI4, PC6; dilatational wave, 2/100 Hz; 30 min before anesthesia till the end of surgery	Sham TEAS	CAM-ICU	Incidence of POD
Guo et al. (2021)	60 (32/28)	Thoracic surgery	TEAS; LI4, PC6; continuous wave, low frequency; intervention time was not reported	Blank control	Unclear	Incidence of POD; duration of POD
Wu et al. (2021)	60 (30/30)	Digestive surgery	TEAS; HT7, PC6, ST36; dilatational wave, 2/100 Hz; 30 min before anesthesia till the end of surgery and three times on the next 24, 48, and 72 h after surgery, postoperative stimulation for 30 min each time	Sham TEAS	CAM	Incidence of POD
Wei et al. (2021)	102 (50/52)	Orthopedic surgery	TEAS; HT7, PC6; continuous wave, 10 Hz; 30 min before anesthesia and at 18:00 on the surgery day and following 2 days after surgery, for a total of four times, 30 min each time	Sham TEAS	CAM	Incidence of POD

TEAS, transcutaneous electrical acupoint stimulation; CAM, confusion assessment method; POD, postoperative delirium; CAM-CR, confusion assessment method-Chinese version; CAM-ICU, confusion assessment method for the intensive care unit; 4AT, 4 A’s test.

### 3.3. TEAS regimen and control interventions

Of the 12 included trials, three trials adopted preoperative TEAS (Qian et al., 2018; He and Sheng, 2020; Ding et al., 2022), three trials adopted intraoperative TEAS (Liu, 2019; Ding et al., 2022; Du et al., 2022), four trials adopted preoperative and intraoperative TEAS (Gao et al., 2018; Ding et al., 2020; Wei et al., 2022; Xu et al., 2022), one used preoperative and postoperative TEAS (Wei et al., 2021), and one used preoperative, intraoperative and postoperative TEAS (Wu et al., 2021). As we can see, the dilatational wave and continuous wave are commonly used. For stimulation frequency, 2/100, 2/30, and 2 Hz were popular among the trials. The most commonly used traditional acupoints, used in three or more trials, were PC6 (*Neiguan*), LI4 (*Hegu*), GV20 (*Baihui*), and ST36 (*Zusanli*).

Five trials used blank control (Qian et al., 2018; He and Sheng, 2020; Guo et al., 2021; Wei et al., 2022; Xu et al., 2022), and seven trials used sham TEAS as a control (Gao et al., 2018; Liu, 2019; Ding et al., 2020, 2022; Wei et al., 2021; Wu et al., 2021; Du et al., 2022). Of these seven trials, six attached electrodes on the same acupoints, but did not administer electrical stimulation (Gao et al., 2018; Liu, 2019; Ding et al., 2020, 2022; Wei et al., 2021; Du et al., 2022), and the remaining one stimulated at sites not corresponding to traditional acupoints (Wu et al., 2021).

### 3.4. Risk of bias

**Figure 2:**
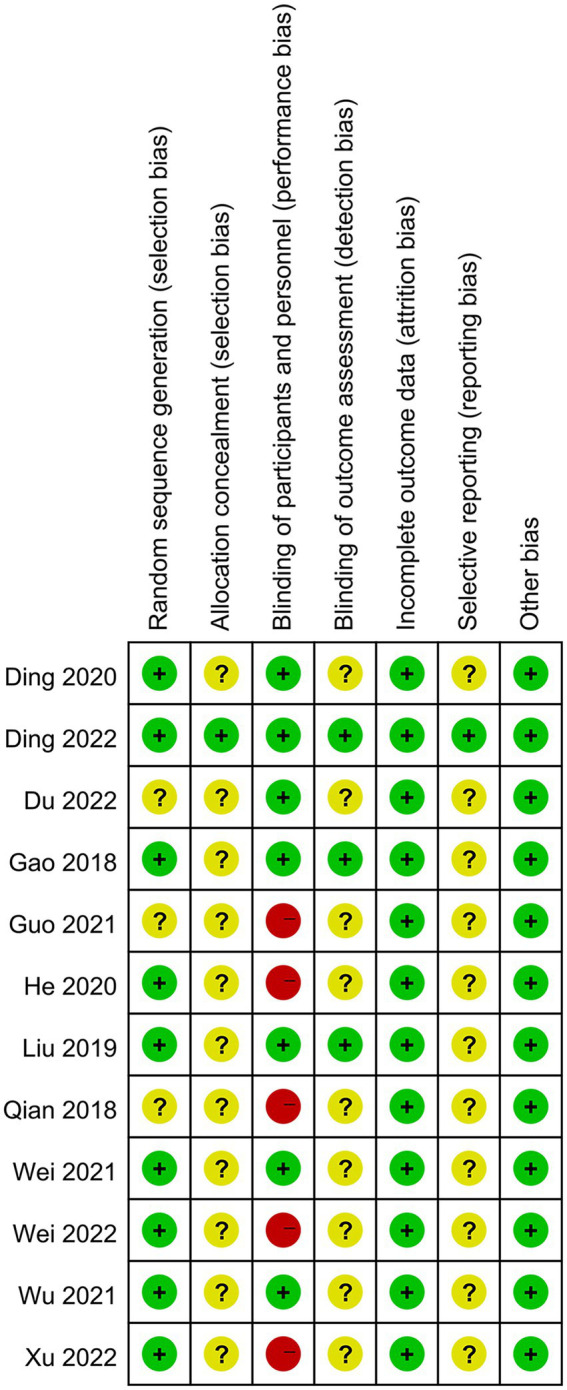
Risk of bias summary.

The risk of bias assessment is shown in Figure 2 and Supplementary Figure S1. Nine trials described the detailed methods of randomization (Gao et al., 2018; Liu, 2019; Ding et al., 2020, 2022; He and Sheng, 2020; Wei et al., 2021, 2022; Wu et al., 2021; Xu et al., 2022), and the remaining three just mentioned randomization (Qian et al., 2018; Guo et al., 2021; Du et al., 2022). Only one trial emphasized allocation concealment (Ding et al., 2022). Seven trials performed a blinding method on subjects and operators (Gao et al., 2018; Liu, 2019; Ding et al., 2020, 2022; Wei et al., 2021; Wu et al., 2021; Du et al., 2022), and three trials described a blinding method on outcome assessments (Gao et al., 2018; Liu, 2019; Ding et al., 2022). All trials with case dropout reported detailed reasons. Only one trial was conducted according to protocols (Ding et al., 2022). The remaining trials’ protocols were not available that made it difficult to judge reporting bias (Gao et al., 2018; Qian et al., 2018; Liu, 2019; Ding et al., 2020; He and Sheng, 2020; Guo et al., 2021; Wei et al., 2021, 2022; Wu et al., 2021; Du et al., 2022; Xu et al., 2022).

### 3.5. Effects on the incidence of POD

**Figure 3:**
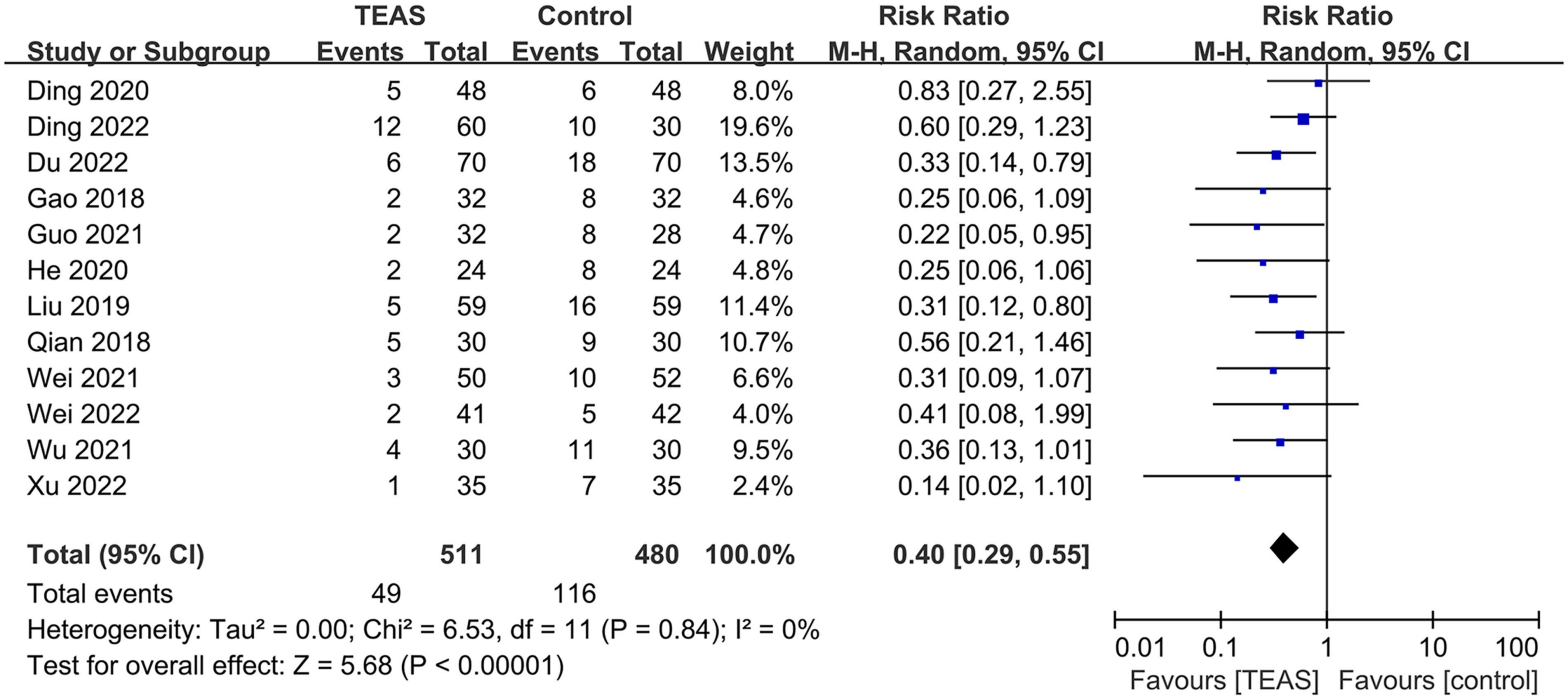
Forest plot of the incidence of POD.

All included trials reported the incidence of POD. The total incidence of POD in TEAS and control groups were, respectively, 9.6% (49/511) and 24.2% (116/480). The meta-analysis showed that TEAS considerably decreased the incidence of POD (*n* = 991; RR = 0.40, 95%CI = 0.29 to 0.55, *p* < 0.00001, Figure 3), with low heterogeneity across trials that reported these findings (*I^2^* = 0%, *p* = 0.84).

**Figure 4:**
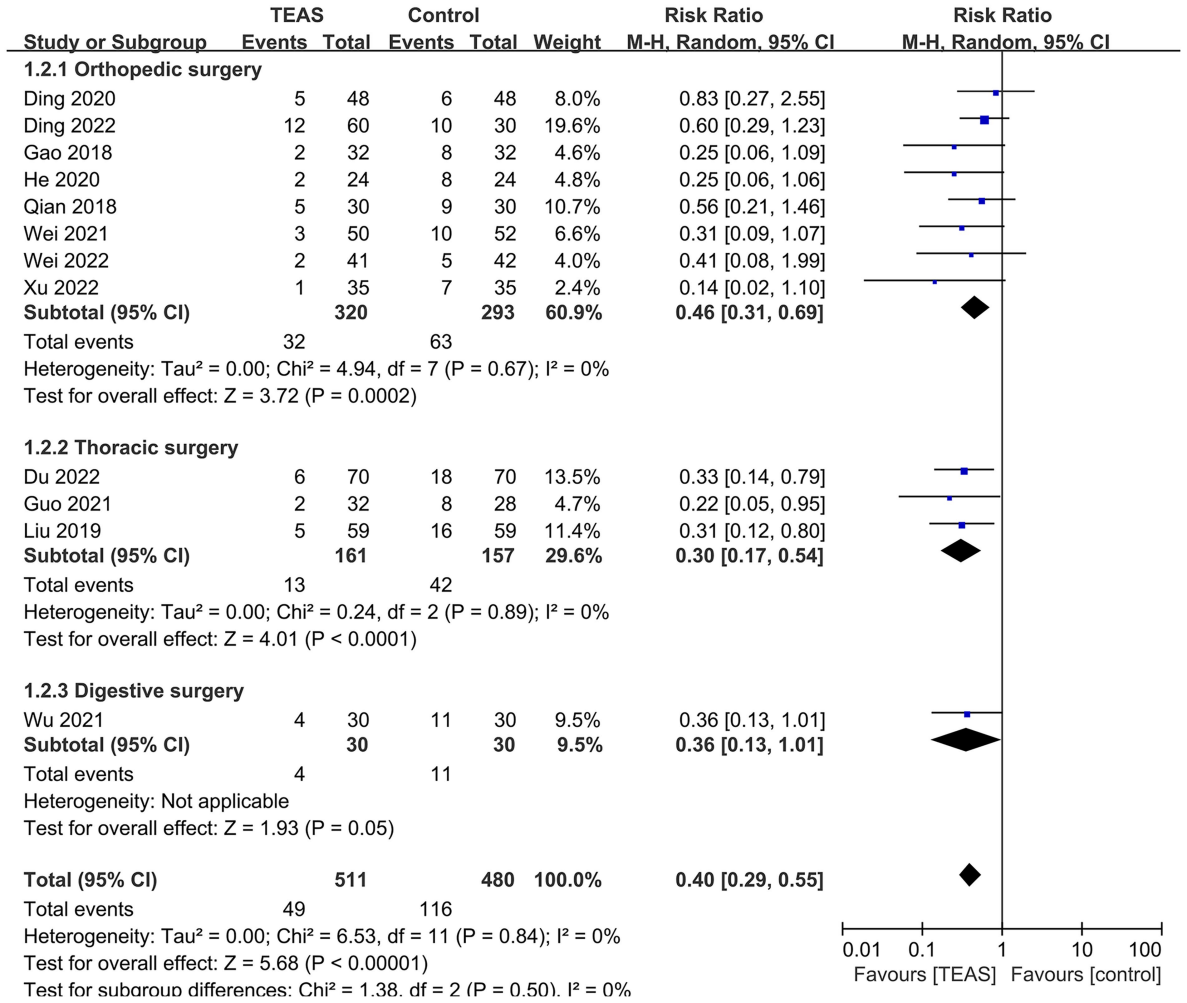
Subgroup analysis of the incidence of POD based on different types of surgery.

**Figure 5:**
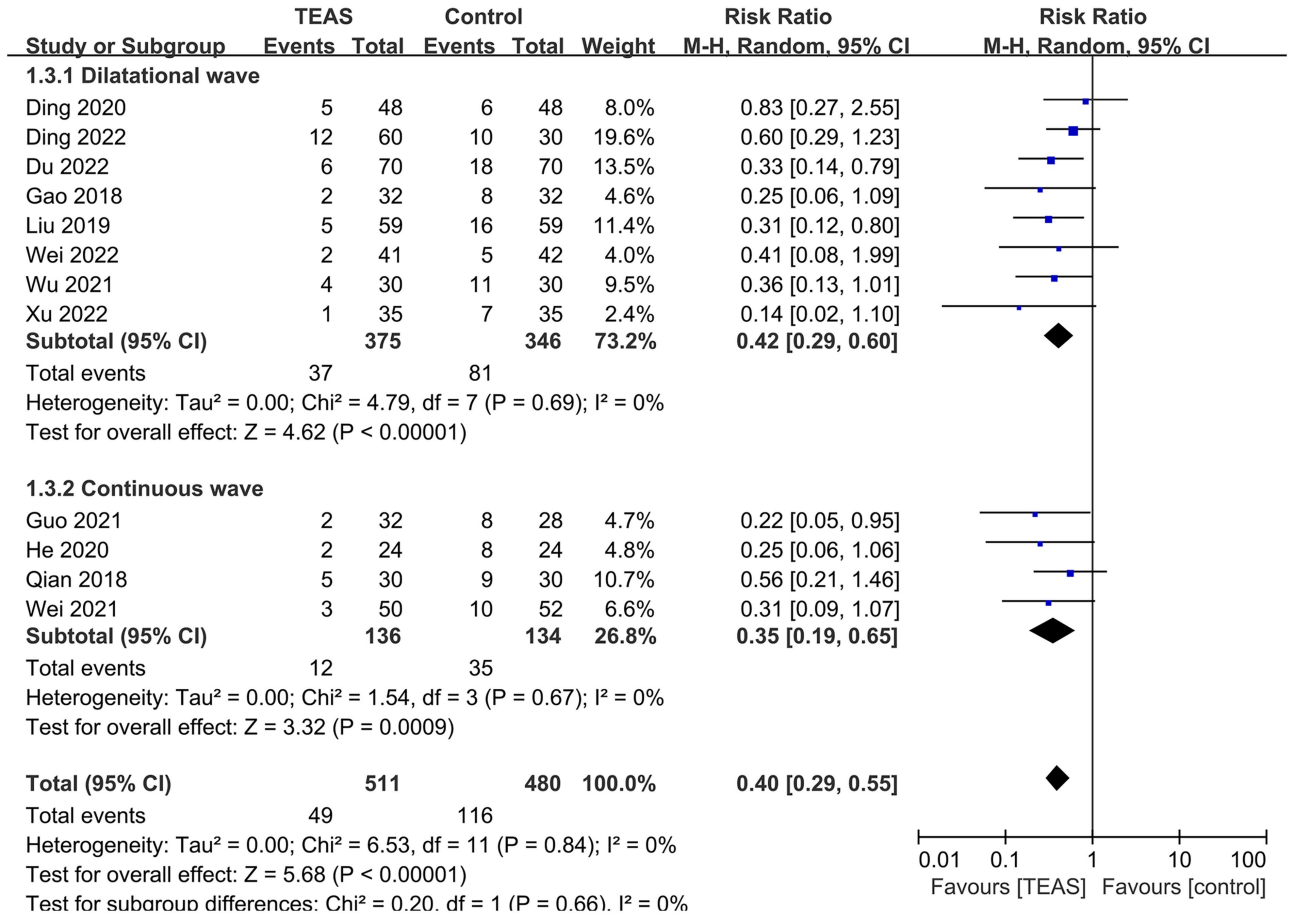
Subgroup analysis of the incidence of POD based on different TEAS waveform.

**Figure 6:**
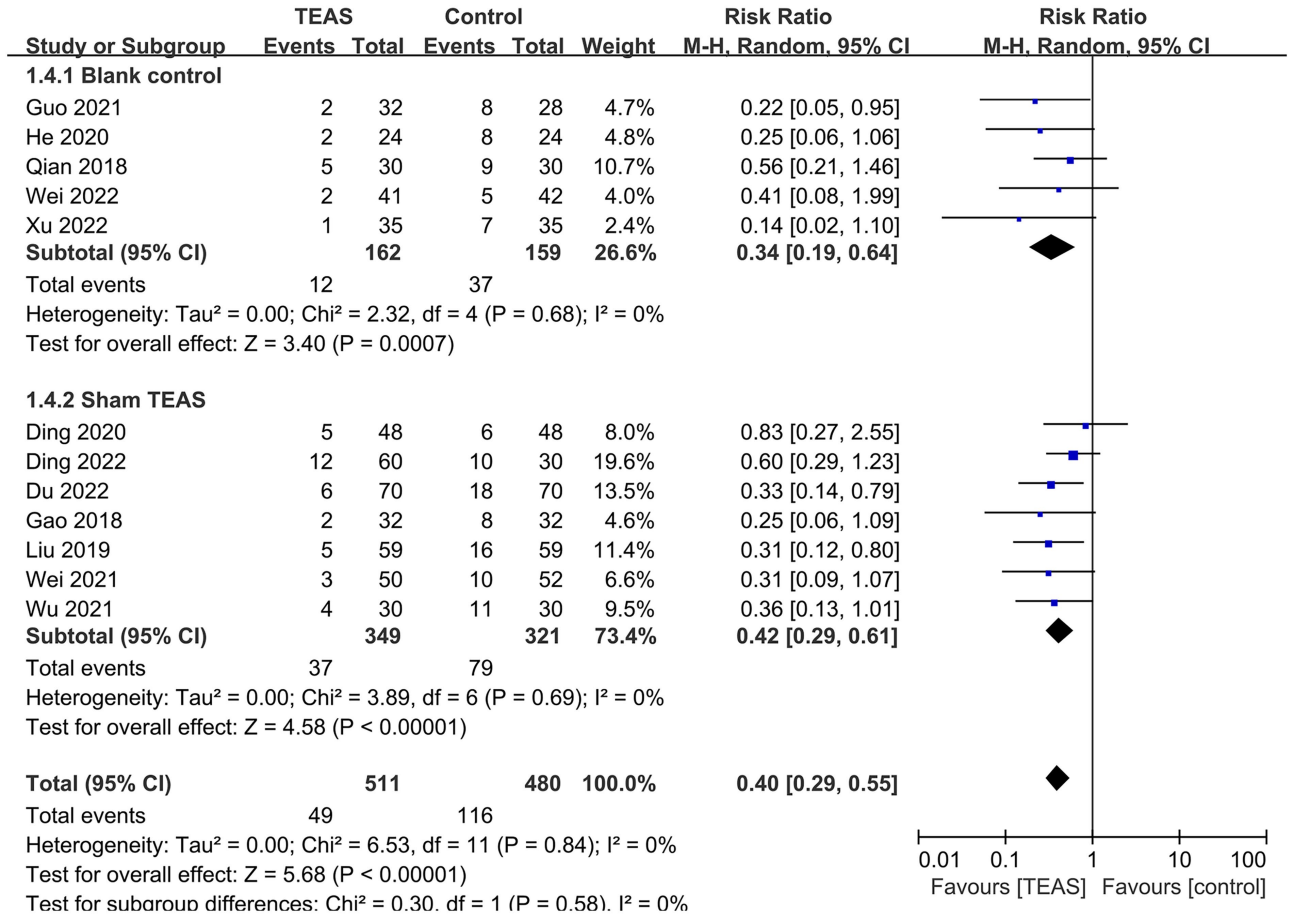
Subgroup analysis of the incidence of POD based on different control interventions.

Subgroup analyses based on different types of surgery (Figure 4), TEAS waveform (Figure 5) and control interventions (Figure 6) were conducted to find potential sources of heterogeneity and evaluate the risk factors influencing POD. Figure 4 showed that TEAS reduced the incidence of POD in elderly patients undergoing orthopedic surgery (RR = 0.46, 95%CI = 0.31 to 0.69, *p* = 0.0002) and thoracic surgery (RR = 0.30, 95%CI = 0.17 to 0.54, *p* < 0.0001), but not in elderly patients undergoing digestive surgery (RR = 0.36, 95%CI = 0.13 to 1.01, *p* = 0.05). Figure 5 showed TEAS with dilatational wave (RR = 0.42, 95%CI = 0.29 to 0.60, *p* < 0.00001) and TEAS with continuous wave (RR = 0.35, 95%CI = 0.19 to 0.65, *p* = 0.0009) were both beneficial. Figure 6 showed that TEAS was favored in preventing POD when compared to blank control (RR = 0.34, 95%CI = 0.19 to 0.64, *p* = 0.0007) and sham TEAS (RR = 0.42, 95%CI = 0.29 to 0.61, *p* < 0.00001).

### 3.6. Effects on the duration of POD

**Figure 7:**
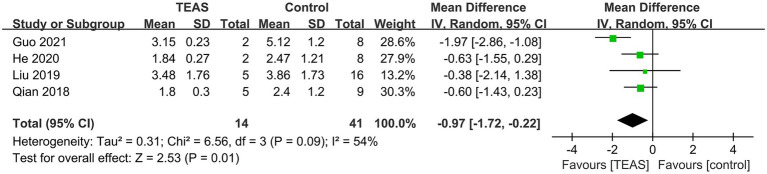
Forest plot of the duration of POD.

Four RCTs reported the duration of POD (Qian et al., 2018; Liu, 2019; He and Sheng, 2020; Guo et al., 2021). The meta-analysis showed a significant difference between TEAS and control groups (*n* = 55; MD = –0.97 days, 95%CI = −1.72 to −0.22, *p* = 0.01, Figure 7), with significant heterogeneity (*I^2^* = 54%, *p* = 0.09).

### 3.7. Publication bias

**Figure 8:**
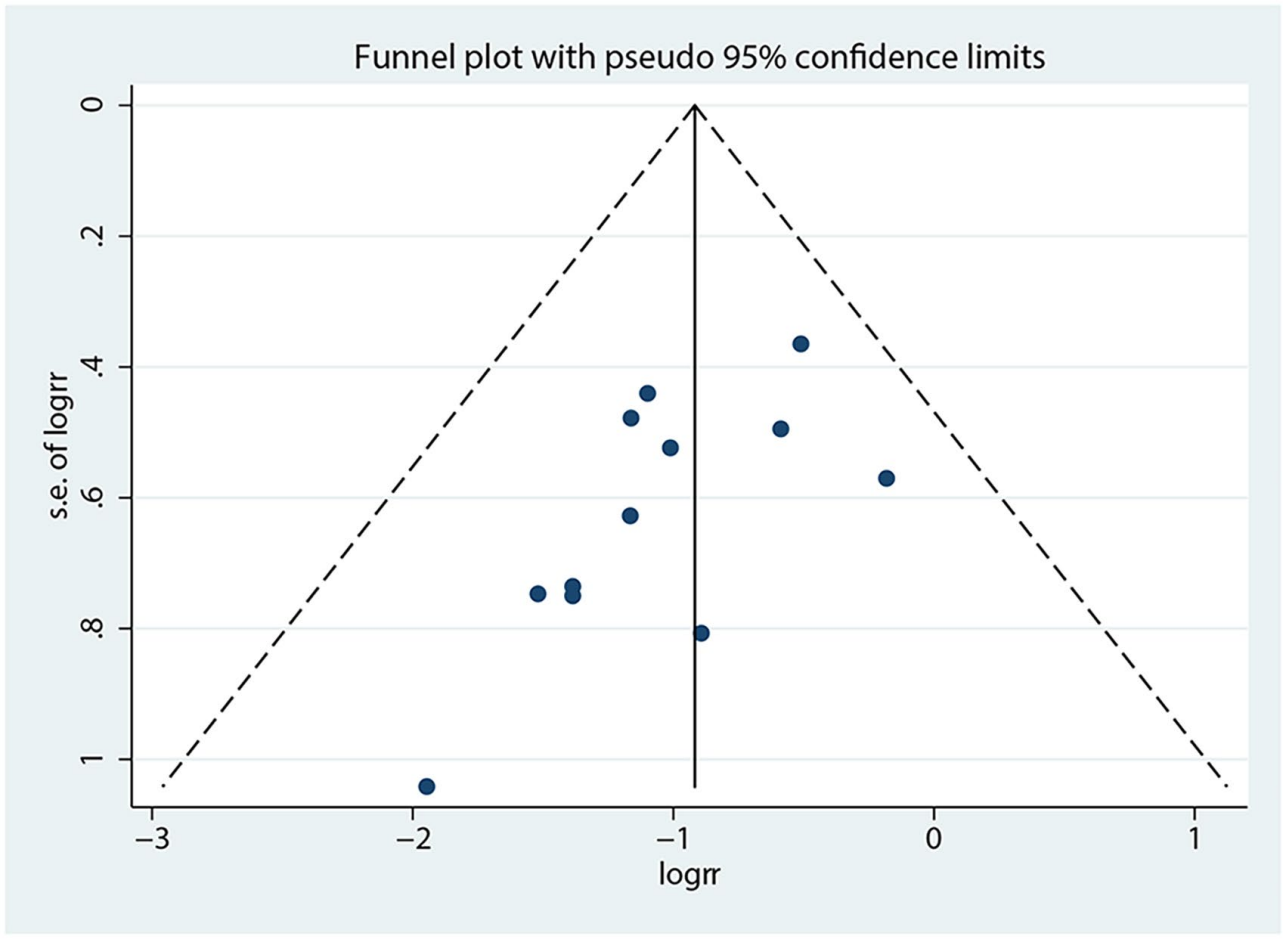
Funnel plot of the incidence of POD.

A funnel plot was used to assess publication bias based on the incidence of POD (Figure 8). Though this funnel plot was visually symmetric, it was hard to rule out the existence of publication bias due to the limited number of trials included for analysis, with Egger’s test (*p* = 0.04).

## 4. Discussion

### 4.1. Summary of main findings

To our knowledge, this is the first systematic review and meta-analysis evaluating the effect of TEAS on the prevention of POD in elderly surgical patients. Twelve RCTs with a total of 991 participants were included. The meta-analysis indicated that TEAS could significantly reduce the incidence of POD and shorten the duration of POD. In view of the fact that the incidence of POD is correlated with type of surgery, subgroup analyses based on different types of surgery were conducted to find potential sources of heterogeneity and evaluate the risk factors influencing POD. The results showed that TEAS could reduce the incidence of POD in elderly patients undergoing orthopedic surgery and thoracic surgery, but not digestive surgery. Of note, only one study involving digestive surgery was included in this outcome. Subgroup analyses based on different waveform showed that both TEAS with dilatational wave and TEAS with continuous wave had significant preventive effect on POD. In TEAS RCTs, inclusion of a sham administration control is necessary to exclude placebo effects. The common approaches to sham TEAS were non-electrical stimulation on the same acupoints and electrical stimulation at sites not corresponding to traditional acupoints. In this review, seven trials adopted sham TEAS (Gao et al., 2018; Liu, 2019; Ding et al., 2020, 2022; Wei et al., 2021; Wu et al., 2021; Du et al., 2022). Subgroup analyses showed that TEAS did have a significant effect on the incidence of POD when compared with blank control and sham TEAS, which emphasized the preventive effect of TEAS on POD in elderly patients. However, the findings should be interpreted with caution because of the restricted number, high risk of bias and potential publication bias of included trials.

Transcutaneous electrical acupoint stimulation, known as acupuncture-like transcutaneous electrical nerve stimulation, is an ideal combination of acupoints and bioelectricity (Francis and Johnson, 2011). Compared with traditional acupuncture, TEAS may be more acceptable to elderly surgical patients. As a non-invasive therapy, TEAS provides similar effects of electroacupuncture and largely avoids the risk of infection. It is user-friendly and can be applied by medical personnel with minimal training. Moreover, acupoints selection and TEAS parameters (waveform, frequency, intensity and intervention time) setting are easy to report and repeat, which makes the repeatability and reliability of TEAS in clinical practice and research better than that of traditional acupuncture. While traditional acupuncture should be operated by professional acupuncturists. The needles cannot be well protected during the procedure, and the overall effects may be minimized.

Regarding the selection of TEAS acupoints, PC6 (*Neiguan*) and LI4 (*Hegu*) were the most commonly used acupoints in these included trials. According to traditional Chinese medicine, PC6 (*Neiguan*) and LI4 (*Hegu*) are important acupoints for analgesia, and PC6 (*Neiguan*) is an important acupoint for neurocognitive dysfunction. These theories have been proved in various clinical trials (Wang et al., 2014) and animal experiments (Yoo et al., 2011). Existing studies have shown that stimulation of PC6 (*Neiguan*) and LI4 (*Hegu*) can be used to assist anesthesia, reduce anesthetic use, repress surgical stress, and improve cognitive function (Wang et al., 2014; Matsumoto-Miyazaki et al., 2017). Its mechanism is related to improving cerebral blood flow and brain metabolism, lowering the permeability of the blood–brain barrier, and reducing neuroinflammation and nerve cell damage. Because pain and sleep disorders are possible confounders of delirium severity, stimulation of PC6 (*Neiguan*) and LI4 (*Hegu*) might reduce the incidence of POD by attenuating the extent of postoperative pain and improving sleep quality (Fan et al., 2022). Regrettably, the question of which specific acupoints, TEAS frequency, intensity, and intervention time are most beneficial could not been solved because of the different combinations of acupoints, the different stimulation parameters, and the limited number of included studies. Therefore, we only performed a meta-analysis of TEAS as a whole and came to a conclusion.

### 4.2. Mechanisms of TEAS

The potential mechanism of POD is related to inflammatory response and alteration in neurotransmitters (Wang and Shen, 2018). Modern studies suggest that TEAS can reduce narcotics consumption, hinder the activation of microglia, reduce oxidative stress, restrain central and peripheral inflammatory reactions, reduce the level of inflammatory factors, lower the permeability of blood–brain barrier and alleviate central nervous system injury to reduce the incidence of POD and shorten the duration of POD (Ding et al., 2022). It has shown that TEAS reduced the values of S100β at the end of surgery and 1 day after surgery, and the values of IL-6 on the 1st postoperative day (Gao et al., 2018). TEAS could reduce hippocampal neurons apoptosis by increasing the Bcl-2/Bax ratio and inhibiting activated caspase-3 expression, and improve mitophagy by upregulating PINK1, Parkin, and LC3-II expression and downregulating LC3-I and p62 expression (Hu et al., 2022). Similar to acupuncture, TEAS may also improve cerebral blood flow and brain metabolism, alleviate perioperative hypotension, reduce anesthetic and analgesic consumption, and improve sleep disorders (Ho et al., 2020; Zhang et al., 2022). Thus, it has been proposed to be used for the treatment of various kinds of postoperative neurological disorders, including POD.

### 4.3. Strengths and limitations

Several strengths of this review were listed below. It is the first published systematic review and meta-analysis to comprehensively investigate the effect of TEAS on the incidence of POD and the duration of POD in elderly surgical patients. Subgroup analyses based on different types of surgery, TEAS waveform and control interventions were conducted to reduce potential heterogeneity and evaluate the risk factors influencing POD. Moreover, a protocol was registered with INPLASY in advance, rendering the potential reporting bias of this review as low.

Several important limitations of this review were as follows. Firstly, the limited number of high-quality trials is a critical limitation. Most included trials lacked details of allocation concealment. Nearly half of the trials did not perform blinding of personnel and participants, and only a small number of trials described a blinding method on outcome assessments. The existence of publication bias is hard to be ruled out due to the limited number of trials included. Secondly, all included trials were conducted in China, which may contribute to region bias. Thirdly, significant heterogeneity was observed when investigating the effect of TEAS on the duration of POD, and the results of the random-effects model were not reliable. Fourthly, the question of which specific acupoints, TEAS frequency, intensity, and intervention time are most beneficial has not been analyzed. Though PC6 (*Neiguan*) and LI4 (*Hegu*) were the two most frequently used acupoints, not any included studies selected PC6 (*Neiguan*) or LI4 (*Hegu*) as the only TEAS acupoint. So we could not analyze the effects of TEAS at PC6 (*Neiguan*) or LI4 (*Hegu*) on POD in elderly surgical patients. Other TEAS parameters, such as TEAS frequency and intervention time, could not be analyzed systematically due to the limited number of included articles.

### 4.4. Implications

Implications for clinical practice are described below. Two guidelines on POD published by European Society of Anesthesiology and American Geriatrics Society both recommended non-pharmacologic interventions for preventing POD in elderly patients (American Geriatrics Society, 2015; Aldecoa et al., 2017). But TEAS was not recommended in these two clinical guidelines. Because of the non-invasive and user-friendly characteristics of TEAS and the positive results of this meta-analysis, we cautiously recommend perioperative TEAS as a routine means of POD prevention in elderly patients. To optimize the effect, PC6 (*Neiguan*), LI4 (*Hegu*), GV20 (*Baihui*), and ST36 (*Zusanli*) can be considered for selection.

Implications for future research are described in the following content. In future, RCTs should be conducted and reported according to the Consolidated Standards of Reporting Trials (CONSORT) statement and the Standards for Reporting Interventions in Clinical Trials of Acupuncture (STRICTA) guideline to improve the methodological quality, report quality and repeatability. The optimal acupoint prescriptions, waveform, frequency, retention period, and total sessions of TEAS are needed to be tested in future. Moreover, high-quality, large sample and multicenter RCTs should be carried out to further determine the reliability of TEAS for POD.

## 5. Conclusion

This systematic review and meta-analysis suggested that TEAS could reduce the incidence of POD and shorten the duration of POD in elderly surgical patients. The findings should be interpreted with caution because of the restricted number and low methodological quality of included trials. High-quality, large sample, and multi-center trials are needed to further confirm the preliminary findings.

## Data availability statement

The original contributions presented in the study are included in the article/Supplementary material, further inquiries can be directed to the corresponding author.

## Author contributions

K-YH, SL, and LC contributed to conception and design of the study. K-YH and SL searched the databases, evaluated eligible articles for inclusion, and performed the statistical analysis. K-YH wrote the first draft of the manuscript. SL, LC, Y-YX, and AG wrote sections of the manuscript. All authors contributed to manuscript revision, read, and approved the submitted version.

## Funding

This work was supported by NINGBO Medical & Health Leading Academic Discipline Project (No. 2022-ZF01), Zhejiang Province Famous Traditional Chinese Medicine Expert Inheritance Studio Construction Project (No. GZS2020038), National TCM Perponderant Specialty Project, NINGBO Medical & Health Brand Discipline Project (No. PPXK2018-07), and the Key Project of Ningbo Natural Science Foundation (No. 2022J279).

## Conflict of interest

The authors declare that the research was conducted in the absence of any commercial or financial relationships that could be construed as a potential conflict of interest.

## Publisher’s note

All claims expressed in this article are solely those of the authors and do not necessarily represent those of their affiliated organizations, or those of the publisher, the editors and the reviewers. Any product that may be evaluated in this article, or claim that may be made by its manufacturer, is not guaranteed or endorsed by the publisher.

## Supplementary material

The Supplementary material for this article can be found online at: https://www.frontiersin.org/articles/10.3389/fnagi.2023.1046754/full#supplementary-material
