# Supplementary material for: Transcutaneous electrical acupoint stimulation for the prevention of postoperative delirium in elderly surgical patients: A systematic review and meta-analysis
Source: Front Aging Neurosci. 2023 Jan 31;15:1046754. doi: 10.3389/fnagi.2023.1046754 (PMC9928205; doi:10.3389/fnagi.2023.1046754)
Supplement: Supplementary file 1 [file Table_1.DOCX]

Supplementary Material

**Appendix 1.** The detailed search strategy.

**Search strategy for CNKI**

(TKA=‘经皮穴位电刺激’+‘经皮’+‘体表’+‘穴位’+‘穴’+‘电刺激’+‘电针’) AND (TKA= ‘谵妄’+‘术后谵妄’+‘谵妄发作’+‘急性脑综合征’)

**Search strategy for WanFang**

主题: ("经皮穴位电刺激" OR "经皮" OR "体表" OR "穴位" OR "穴" OR "电刺激" OR "电针") and 主题: ("谵妄" OR "术后谵妄" OR "谵妄发作" OR "急性脑综合征")

**Search strategy for PubMed**

| Search | Terms |
| --- | --- |
| #1 | "acupuncture"[MeSH Terms] |
| #2 | "acupuncture point"[MeSH Terms] |
| #3 | "acupuncture therapy"[MeSH Terms] |
| #4 | "transcutaneous electrical acupoint stimulation"[Title/Abstract] OR "TEAS"[Title/Abstract] OR "transcutaneous acupoint electrical stimulation"[Title/Abstract] OR "TAES"[Title/Abstract] OR "acustimulation"[Title/Abstract] OR "acupuncture point"[Title/Abstract] OR "acupoint"[Title/Abstract] OR "electroacupuncture"[Title/Abstract] OR "electric"[Title/Abstract] OR "acupuncture"[Title/Abstract] |
| #5 | "delirium"[MeSH Terms] |
| #6 | "postoperative delirium"[Title/Abstract] OR "delirium"[Title/Abstract] OR "delirium episodes"[Title/Abstract] OR "acute brain syndrome"[Title/Abstract] |
| #7 | "randomized controlled trial"[Publication Type] |
| #8 | "clinical trial"[Publication Type] |
| #9 | random*[Title/Abstract] OR controlled[Title/Abstract] OR trial[Title/Abstract] |
| #10 | #1 OR #2 OR #3 OR #4 |
| #11 | #5 OR #6 |
| #12 | #7 OR #8 OR #9 |
| #13 | #10 AND #11 AND #12 |

**Search strategy for CENTRAL**

| Search | Terms |
| --- | --- |
| #1 | (transcutaneous electrical acupoint stimulation):ti,ab,kw |
| #2 | (TEAS):ti,ab,kw |
| #3 | (transcutaneous acupoint electrical stimulation):ti,ab,kw |
| #4 | (TAES):ti,ab,kw |
| #5 | (acustimulation):ti,ab,kw |
| #6 | (acupuncture point):ti,ab,kw |
| #7 | (acupoint):ti,ab,kw |
| #8 | (electroacupuncture):ti,ab,kw |
| #9 | (electric):ti,ab,kw |
| #10 | (acupuncture):ti,ab,kw |
| #11 | MeSH descriptor: [Delirium] explode all trees |
| #12 | #1 OR #2 OR #3 OR #4 OR #5 OR #6 OR #7 OR #8 OR #9 OR #10 |
| #13 | #11 AND #12 |
