# Supplementary figures and images for: Transcutaneous electrical acupoint stimulation for the prevention of postoperative delirium in elderly surgical patients: A systematic review and meta-analysis
Source: Front Aging Neurosci. 2023 Jan 31;15:1046754. doi: 10.3389/fnagi.2023.1046754 (PMC9928205; doi:10.3389/fnagi.2023.1046754)

## *Supplementary Material*

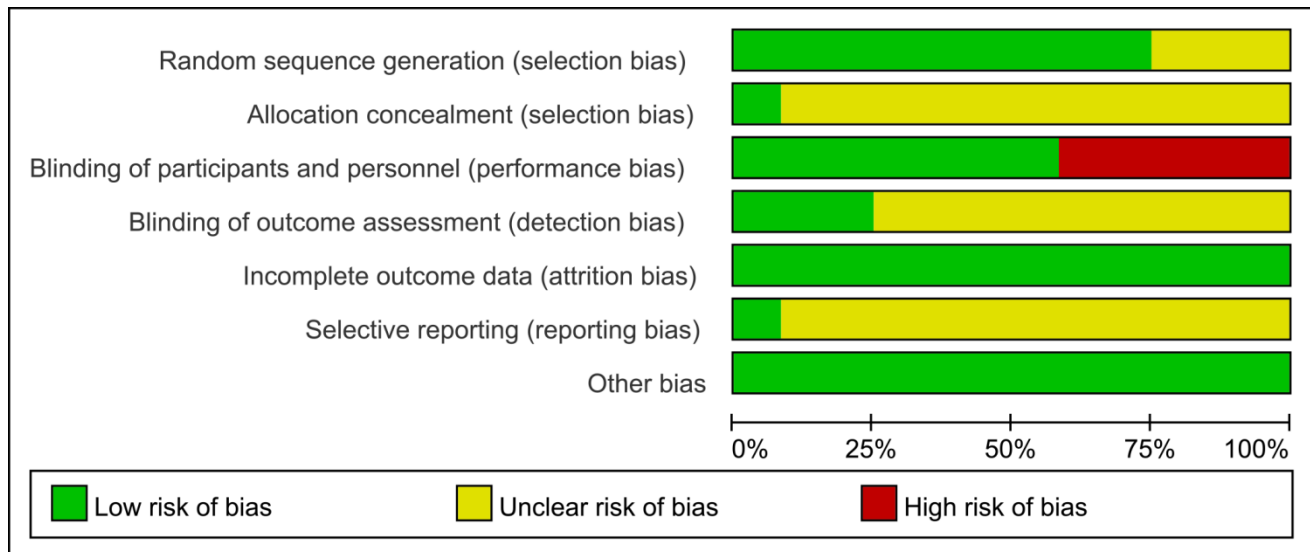

**Supplementary Figure 1.** Risk of bias graph.

Supplement: Supplementary file 2 [file Image_1.pdf]
